# Adversarial bandit approach for RIS-aided OFDM communication

**DOI:** 10.1186/s13638-022-02184-6

**Published:** 2022-11-17

**Authors:** Messaoud Ahmed Ouameur, Lê Dương Tuấn Anh, Daniel Massicotte, Gwanggil Jeon, Felipe Augusto Pereira de Figueiredo

**Affiliations:** 1 Department of Electrical and Computer Engineering, Université du Québec à Trois-Rivières, 3351 Boul des Forges, Trois-Rivières, QC G9A 5H7 Canada; 2 Faculty of Information Technology, VNU-HCM University of Science, Ho Chi Minh City, Vietnam; 3 Department of Embedded Systems Engineering, College of Information Technology, Incheon National University, Incheon, South Korea; 4 National Institute of Telecommunications, Santa Rita Do Sapucaí, Minas Gerais Brazil

**Keywords:** Reconfigurable intelligent surfaces, Reflection beamforming prediction, Deep learning, Machine learning, Sixth-generation (6G) wireless systems, Adversarial bandit, Exponential-weight algorithm for exploration and exploitation, Follow the perturbed leader (FPL)

## Abstract

To assist sixth-generation wireless systems in the management of a wide variety of services, ranging from mission-critical services to safety-critical tasks, key physical layer technologies such as reconfigurable intelligent surfaces (RISs) are proposed. Even though RISs are already used in various scenarios to enable the implementation of smart radio environments, they still face challenges with regard to real-time operation. Specifically, high dimensional fully passive RISs typically need costly system overhead for channel estimation. This paper, however, investigates a semi-passive RIS that requires a very low number of active elements, wherein only two pilots are required per channel coherence time. While in its infant stage, the application of deep learning (DL) tools shows promise in enabling feasible solutions. We propose two low-training overhead and energy-efficient adversarial bandit-based schemes with outstanding performance gains when compared to DL-based reflection beamforming reference methods. The resulting deep learning models are discussed using state-of-the-art model quality prediction trends.

## Introduction

Sixth-generation (6G) wireless systems are expected to enable greater levels of autonomy, improve human–machine interfacing, and achieve deep connectivity in more diverse environments. To assist 6G in managing a wide variety of services, ranging from mission-critical services (e.g., autonomous driving) to safety-critical tasks (e.g., remote surgery), key enabling physical layer technologies (PHY) such as ultra-massive multiple-input multiple-output (MIMO) systems, millimeter wave and Tera-Hertz communications, and reconfigurable intelligent surfaces (RISs), need to be carefully designed [1]. Unfortunately, current network design practices conform to a hypothesis that regard the wireless environment between communicating devices to be unmodified and which can be only overcome through the design of advanced transmission and reception schemes. Breaking free from such a hypothesis by programming the environment is expected to enable major performance gains. As such, RIS-aided communication has received increasing interest from the research community due to its potential in extending the coverage, enhancing link quality and capacity, and mitigating interference and security breaches [2]. RISs enable the reconfiguration of the wireless propagation environment by intelligently controlling the signal reflections via their massive low-cost elements. By jointly adapting the reflected signal amplitude and/or phase shift at each RIS element based on the wireless channels, the signals reflected by the RIS can be constructively combined at the intended receiver. Unlike traditional active relaying/beamforming techniques, RIS is designed to be totally or nearly passive, thus enjoying lower hardware cost and energy consumption [1]. So far, RIS has been adopted in various scenarios. In [3], the error performance of an RIS-aided single-input single-output (SISO) system is examined; meanwhile, RISs are also used for multi-user systems to maximize the signal-to-interference-plus-noise ratio [4] or to enhance energy efficiency [5]. Unfortunately, due to the additional channel links between the RIS and its associated transmitter and intended receivers, the large gain is achieved at the expense of increased overhead for channel estimation [6]. Early works focus on the design of reflection beamforming coefficients under the assumption of perfect channel state information [7], which helps in deriving the system performance bounds, but the underlying optimal techniques are unfortunately algorithm-deficient. Obtaining this channel knowledge, in practice, may require large and possibly prohibitive training overhead, which represents the main challenge for real-time RIS operation. The authors in [6] have acknowledged the main practical issues in RIS-aided wireless communications, wherein the acquisition of accurate channel state information (CSI) is vital but turns out to be practically challenging due to the lack of active components for baseband signal processing, in addition to the fact that an RIS is typically composed of a huge number of passive elements that potentially have different channel coefficients to be acquired. As such, a substantial increase in the system overhead for RIS channel estimation is expected, unless low complexity signal processing methods such as least square (LS) and linear minimum mean square error (LMMSE) algorithms are used [8]. Under the constraint of limited training time, the authors in [9] has resorted to a joint design of the RIS reflection beamforming vector and transmit pilot sequence. Moreover, the idea of grouping RIS elements to reduce the complexity of the channel estimation process is also introduced in [10] whereas exploiting the quasi-static RIS-Transmitter can further aid in efficiently estimating the dynamic RIS-receiver channel^1^ [11]. It is also recognized that a hybrid channel estimation method over a semi-passive RIS architecture, has the potential of reducing the real-time training overhead more, as compared to their separate approaches used in fully passive RIS architectures [12].

Nevertheless, implementing the RIS using discrete (and possibly non-accurate) phase shifters makes it difficult to analytically model such behavior in a tractable manner, making the overall end-to-end model-deficient. Furthermore, due to several practical aspects such as channel aging and limited feedback overhead, it is hard to obtain perfect CSI in practice [6].

Under such deficiencies, machine learning is introduced and has started to be extensively used to enhance the implementation of various components within the 5G radio access network (RAN) [13]. In addition to the smart radio environment concept, embracing a vision wherein 6G is designed in a way that ML could modify parts of the physical (PHY) and medium access control (MAC) layers is proposed [13]. Deep Learning (DL) has also been used for devising computationally efficient approaches for physical layer communication receiver modules. Under the supervised learning approach, the authors in [14] present a DL framework for MIMO symbol detection. It has been able to achieve near-optimal detection performance with an even faster real-time implementation. A recurrent neural network (RNN)-based detection scheme is introduced in [15] for MIMO orthogonal frequency division multiplexing (OFDM) systems and is shown to outperform traditional detection techniques under channel impairments and hardware nonlinearities. Convolutional neural network (CNN)-based supervised learning techniques can also be utilized for channel estimation problems, providing improved generalization abilities and robustness to channel alterations [16]. A DL-based beam prediction method was proposed for distributed mmWave MIMO systems to cope with highly mobile users with negligible training overhead and high data rate gains [17].

Machine learning approaches for RIS have attracted considerable attention for channel estimation [18–20], resource management [21–23], signal detection [24], joint active and passive beamforming [25] and RIS’s reflection beamforming [26–29]. An up-to-date survey is found in [30]. The authors in [25] addressed the phase shift design from joint active and passive beamforming optimization problems for secure beamforming for MISO systems, MISO uplink communication networks and computation offloading in IoT networks use cases. They have provided a review of the current optimization and artificial-intelligence-based methods for handling the constraints imposed by RISs. So far, most works rely on the unity amplitude assumption, whereas the authors in [31] considered a practical phase-dependent amplitude model in which the RIS reflection amplitudes vary with the discrete phase-shifts. Therefore, to solve the complicated problem of joint relay selection and RIS reflection coefficient optimization, a deep reinforcement learning (DRL) model is used to learn from the environment how to obtain the solution and reduce the computational complexity.

The authors in [8] proposed a novel design of the reflection pattern to aid the channel estimation at the access point (AP) formulated as a non-convex problem P1. The estimated channel is used to optimize the reflection coefficient formulated as problem P2. The success of the method is attributed to overcoming the semi-definite relaxation (SDR) complexity by exploiting the strongest signal path in the time domain. The simulation results demonstrated the effectiveness of the method in frequency selective Rician fading channels. Later in the same year, the authors in [32] proposed a new transmission protocol for wideband RIS-assisted single-input multiple-output (SIMO) OFDM communication systems. In [32], each transmission frame is divided into multiple sub-frames where the associated channel state information over consecutive sub-frames is progressively estimated, based on which the passive beamforming at the RIS is fine-tuned to improve the achievable rate. Even if these works did not consider a deep learning-based approach, they open a new perspective for progressive data collection and online training in the event the new transmission framework is adopted. This aspect is considered as future work as for our simulation purposes, the ray tracing scenario “O1” in [33] is used. This scenario is publicly available in [33] and widely adopted [18, 26, 27, 29].

On the other hand, the authors in [26] present a novel semi-passive RIS hardware architecture where fewer active elements are used to assist in estimating the uplink and downlink CSI associated to these active elements like in a traditional MIMO system. Two solutions based on compressive sensing and deep reinforcement learning with very negligible training overhead have been proposed. The DL approach avoids resorting to explicit CSI estimation for the overall RIS-related channels while directly learning the RIS reflection beamforming vectors. Such an end-to-end RIS has recently been proposed to further alleviate the burden in learning directly all the channel parameters [34, 35]. Deep reinforcement learning (DRL) has also been applied for designing efficient spectrum access [36] and scheduling strategies [37] for cellular networks. Automatic cell-sectorization for cellular network coverage maximization is another area where DRL has shown tremendous potential [38].

In this paper, we focus on a semi-active RIS architecture with a very small number of active elements and we propose two efficient reinforcement learning-based schemes where the main contributions are as follows:

- We propose an adversarial bandit approach based on exponential-weight algorithm for exploration and exploitation (EXP3). To show the merits of the proposed scheme, we conduct extensive simulation using the publicly available accurate ray tracing-based DeepMIMO dataset [39] with the 'O1' scenario. The novelty stems from using the training dataset build from the combined channel and the pull-probability of the reflection beamforming vector (elements of the codebook). The proposed EXP3-based scheme requires substantially less data as compared to the DL reflection beamforming technique, owing to the optimal selection of the dataset which stresses that less likely reflection beams are given lower probability, excluding them during the exploitation phase of the EXP3 algorithm. As such, the proposed scheme requires less training dataset size, lower number of active elements, etc.
- To improve upon the computational complexity, the Follow the Perturbed Leader (FPL) scheme is discussed.
- To compare the quality of the state-action deep neural network models used with the reference methods in [26] and with the prosed ones (EXP3 and FPL), we leverage state-of-the-art techniques such as the power low (PL) exponents [40].

The paper is organized as follows: The system model and problem formulation are presented in Section II. Section II also discusses the proposed adversarial bandit approaches. Section III is devoted to discussing the results in terms of achievable rate and energy efficiency while considering a low complexity alternative using a FPL algorithm. The associated DL models’ quality is also analyzed using PL exponents. Finally, the conclusions are made and future research directions are outlined in Section V.

## Methods

The independent and identically distributed Rayleigh fading channel is not physically present when using RIS with a rectangular arrangement. Therefore, an alternative physically feasible model for evaluating RIS-aided communications is required [41]. To enable practical implementations of RIS-aided communication systems, new path loss models [2, 41], and open-source channel models [2, 39] have been developed. As such to reproduce the results and perform a fair comparison, we will adopt the system and channel model in [26].^2^

### System model

**Fig. 1:**
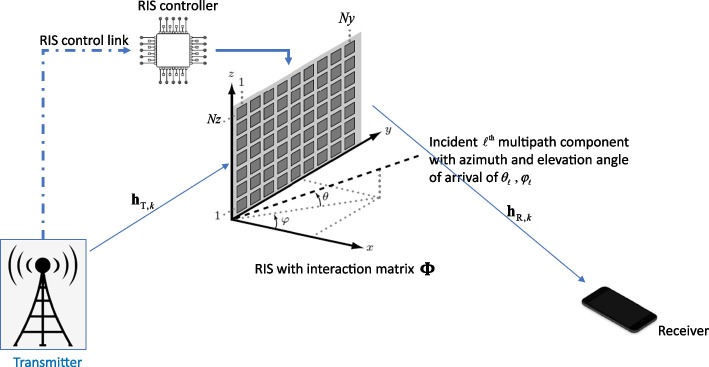
The system model in which transmitter–receiver communication is aided by a RIS having an $M \times M$ interaction matrix where $M = Ny \cdot Nz$

As depicted in Fig. 1, transmitter–receiver communication is aided by an RIS having *M* reconfigurable elements. For the sake of simplicity, we assume that both the transmitter and receiver are equipped with a single antenna. For generalization, one can adopt the signal model from [2]. An OFDM-based transmission with *K* subcarriers is adopted. The links via the RIS are represented by $M \times 1{\kern 1pt}$ complex valued vectors ${\mathbf{h}}_{T,k} ,{\mathbf{h}}_{R,k} \in {\mathbb{C}}^{M \times 1}$. By neglecting the direct path,^3^ the received signal can be written as

1 $$y_{k} = {\mathbf{h}}_{{{\text{R,}}k}}^{T} {\mathbf{\Phi h}}_{{{\text{T,}}k}} s_{k} + n_{k}$$

where ${{\varvec{\Phi}}} \in {\mathbb{C}}^{M \times M}$ is the RIS interaction diagonal matrix, $s_{k}$ and $n_{k}$ are the transmitted symbol per subcarrier *k* and the receive noise with zero mean and variance of $\sigma_{n}$. With $P_{{\text{T}}}$ being the total transmit power, the follow power constraint per subcarrier is enforced $E\left( {\left| {s_{k} } \right|^{2} } \right) = {{P_{{\text{T}}} } \mathord{\left/ {\vphantom {{P_{{\text{T}}} } K}} \right. \kern-\nulldelimiterspace} K}$. Herein $\left( \cdot \right)^{T}$ and $E\left( \cdot \right)$ denote the transpose and the expectation operations, respectively. If we re-arrange the diagonal elements of the interaction matrix ${{\varvec{\Phi}}}$ in an $M \times 1{\kern 1pt}$ column vector ${{\varvec{\uppsi}}}$, we refer to it as the reflection beamforming (BF) vector, such that ${{\varvec{\Phi}}} = {\text{diag}}\left( {{\varvec{\uppsi}}} \right)$, Eq. (1), can also be expressed in more convenient way as

2 $$y_{k} = \left( {{\mathbf{h}}_{R,k} \odot {\mathbf{h}}_{{{\text{T,}}k}} } \right)^{T} {{\varvec{\uppsi}}}s_{k} + n_{k}$$

where $\odot$ denotes the Hadamard product. Imposing few practical implementation constraints of a nearly passive RIS where the phase shifters apply the same phase shift over all subcarriers, the *m*-th element of ${{\varvec{\uppsi}}}$ is modeled as $\left[ {{\varvec{\uppsi}}} \right]_{m} = e^{{j\phi_{m} }}$.^4^

### Channel model and RIS design objective

As for the channel model, a wideband geometric channel model for ${\mathbf{h}}_{{{\text{T}},k}}$ and ${\mathbf{h}}_{{{\text{R}},k}}$ is used [2 26, 41]. Therefore, ${\mathbf{h}}_{{{\text{T,}}k}}$ and ${\mathbf{h}}_{{{\text{R,}}k}}$ is expressed as a function of the azimuth and elevation angles of arrival/departure of the $\ell{{{\text{th}}}}$ path from a total of *L* paths such that the array vector of the RIS is defined as ${\mathbf{a}}\left( {\theta_{\ell } \;,\;\varphi_{\ell } } \right) \in {\mathbb{C}}^{M \times 1\;}$ where $$\theta_{\ell } \in \left[ {\begin{array}{*{20}c} 0 & {2\pi } \\ \end{array} } \right)$$ and $$\varphi_{\ell } \; \in \left[ {\begin{array}{*{20}c} 0 & \pi \\ \end{array} } \right)$$ (see Fig. 1). For the sake of brevity, we refer the reader to [2] and [26] for detailed modeling.

The RIS design objective is therefore to find out the reflection BF vector ${{\varvec{\uppsi}}}$ that maximizes the achievable rate at the receiver

3 $$R = \frac{1}{K}\sum\limits_{k = 1}^{K} {\log_{2} \left( {1 + \rho \left| {\left( {{\mathbf{h}}_{R,k} \odot {\mathbf{h}}_{{{\text{T,}}k}} } \right)^{T} {{\varvec{\uppsi}}}} \right|^{2} } \right)}$$

where the signal-to-noise ratio is $\rho = {{P_{T} } \mathord{\left/ {\vphantom {{P_{T} } {K\sigma_{n} }}} \right. \kern-\nulldelimiterspace} {K\sigma_{n} }}$. The maximization is done over a discrete pre-defined codebook $P$ due to the fact that a practical radio frequency (RF) phase shifter uses quantized phase values. Unfortunately, maximizing (3) entails an exhaustive search over the codebook $P$. Fortunately, the authors in [26] have proposed a novel hardware architecture along with a compressive sensing and DL-based framework to tackle the issue with low training overhead. However, there is still a large room for improvement as we will discuss throughout this paper.

### Proposed algorithm using Adversarial bandit approach via exponential-weight algorithm for exploration and exploitation

The authors in [26] use a DL-based approach to predict the reflection BF vector. Over a channel coherence block size *S*, the RIS receives two pilots to estimate a sampled channel vector ${\overline{\mathbf{h}}}\left( s \right) = {\text{vec}}\left( {\left[ {{\overline{\mathbf{h}}}_{1} \left( s \right),{\overline{\mathbf{h}}}_{2} \left( s \right),{\kern 1pt} \cdots ,{\overline{\mathbf{h}}}_{K} \left( s \right)} \right]} \right)$ where ${\overline{\mathbf{h}}}_{k} \left( s \right) \in {\mathbb{C}}^{{\overline{M} \times 1}}$ denotes the sampled combined channel vector, ${\mathbf{h}}_{{{\kern 1pt} k}} = {\mathbf{h}}_{R,k} \odot {\mathbf{h}}_{{{\text{T,}}k}}$, for the *k*-th subcarrier at *s*-th channel coherent block using a fraction number of the RIS elements $\overline{M} \ll M$ that are assumed to be active elements (i.e., equipped with full RF and baseband processing receiver chain for an effective uplink and downlink channel estimation). During beam training, the RIS is configured using one reflection beam ${{\varvec{\uppsi}}}$(notice that the subscript *k* is removed because one reflection BF vector is available for all subcarriers) from the codebook $P$. Then, a dataset is contracted out of the tuples $\Upsilon \leftarrow \left( {{\overline{\mathbf{h}}}\left( s \right),{\mathbf{r}}\left( s \right)} \right)$ where ${\mathbf{r}}\left( s \right) = \left[ {R_{1} \left( s \right),R_{2} \left( s \right), \cdots ,R_{N} \left( s \right)} \right]^{T}$ and $R_{n} \left( s \right)$ is the measured rate using the *n*-th codebook (*N* is the cardinality of the codebook $P$). Finally, a deep neural network is trained using the dataset $\Upsilon$.

### Adversarial bandit approach via exponential-weight algorithm for exploration and exploitation

Despite the novel architecture that suggests the use of a few active elements to sample the uplink and downlink channel vectors, the proposed algorithm can be substantially improved. As such, we propose an approach based on adversarial bandit scheme wherein instead of spanning equally every element of the codebook $P$, we adopt a scheme that favors the more likely optimal beams. Therefore, the dataset $\Upsilon$ will have more useful data to train with. Table 1 shows the proposed adversarial bandit based on exponential-weight algorithm for exploration and exploitation (EXP3) [42]. The adversarial bandit scheme is a variant of the multi-armed bandit problem where a fixed limited set of resources (phase shifters) must be assigned among alternative choices (reflection beamforming) in a way that maximizes their expected gain (achievable rate), when the properties of each choice are only partly known at the time of assignment and may become better comprehended as time passes. This is one of the strongest generalizations of the bandit problem as it disregards all assumptions of the distribution. In its basic form [43], EXP3 chooses a reflection beamforming vector ${{\varvec{\uppsi}}}_{n}$ (steps 4 and 5 in Table 1) from the codebook $P$ at random with probability $\left( {1 - \gamma } \right)$ where it prefers choices with higher weights (exploit), or it selects with probability $\gamma$ to uniformly randomly explore. After receiving the rewards (steps 6 and 7), the weights are updated (steps 9 and 10). The exponential growth significantly increases the weight of good reflection beamforming vectors.

**Table 1:**
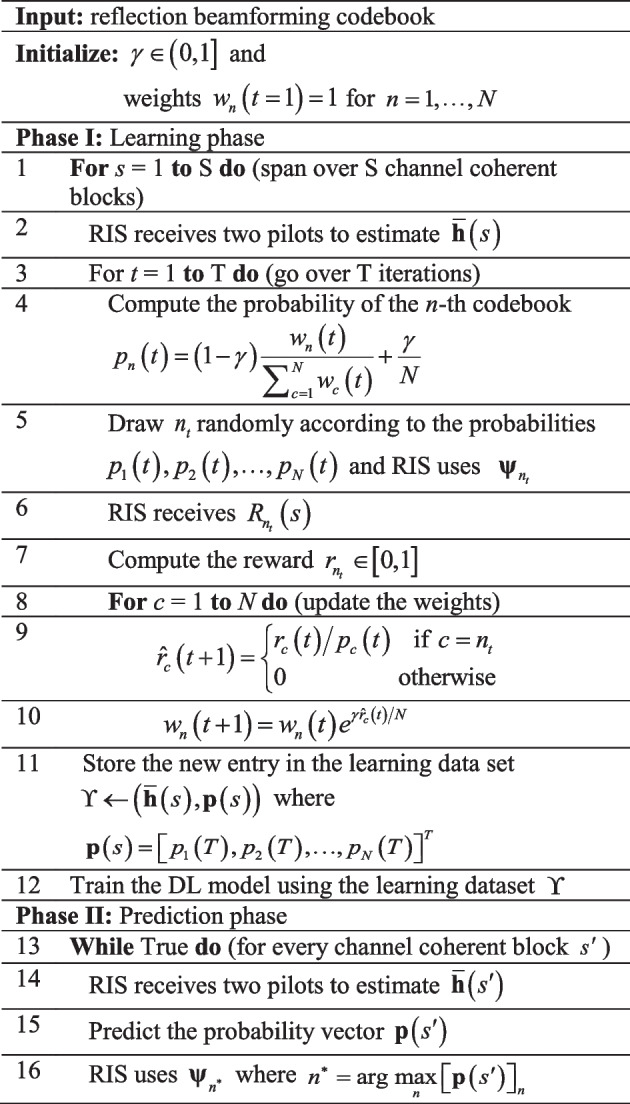
Adversarial bandit-based scheme for reflection beamforming vector perdition

#### A note on the training overhead

**Fig. 2:**
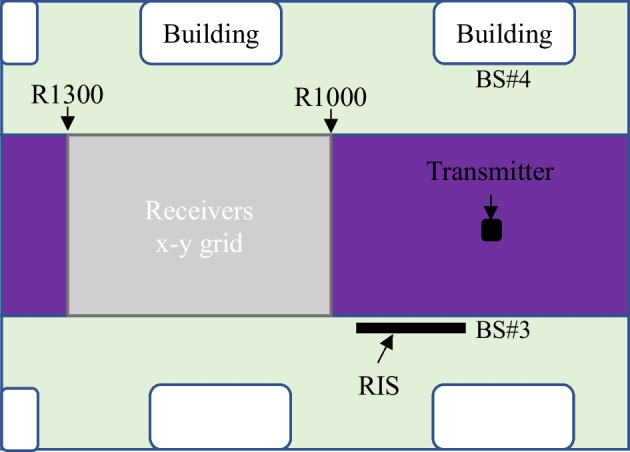
The ray tracing scenario ‘O1’ from [33]. The BS#3 is selected to be the RIS while the transmitter is fixed at the position of raw R850 and column 90. The receiver can be positioned at any 54,300 points within the x–y grid between raw R1000 and R1300. These points constitute the dataset which is split into 80% training set and 20% test set

Over one coherent block *s*, steps 4 to 10 are repeated *T* times where *T* is the number of the EXP algorithm iterations which set to the size of the codebook $N = \left| P \right|$ (future considered works are to study the optimal number of iterations subject to varying system parameters). In every iteration one reflection beamforming vector is selected from a codebook based on EXP algorithm (step 5). This is used in the subsequent pilot transmission where the receiver computes the corresponding achievable rate (3). The achievable rate is used to compute the reward (steps 7–9) and then updates the associated weight (step 10). These weights are in turn used to compute the probability $p_{n} \left( t \right)$ of the selected reflection beamforming vector (step 4). After *T* iterations the dataset is updated as $\Upsilon \leftarrow \left( {{\overline{\mathbf{h}}}\left( s \right),{\mathbf{p}}\left( s \right)} \right)$ where ${\mathbf{p}}\left( s \right) = \left[ {p_{1} \left( T \right),p_{2} \left( T \right), \ldots ,p_{N} \left( T \right)} \right]^{T}$ which depicts the probability of every element of the codebook for the coherent block *s*. The process is repeated over *S* coherent blocks which corresponds to 54,300 possible positions from the ray tracing scenario ‘O1’ (see Fig. 2) used in our simulations. 80% of the training dataset $\Upsilon$ is used to train a deep neural network (DNN) to learn the mapping between the combined channel vector and the probability of the elements of the codebook. During the detection phase (normal operation), only two pilots are transmitted per coherent block $s^{\prime}$. The combined channel vector ${\overline{\mathbf{h}}}\left( {s^{\prime}} \right)$ is computed using the low number of active elements and fed to the DNN to infer the probability of the elements of the codebook ${\mathbf{p}}\left( {s^{\prime}} \right) = \left[ {p_{1} ,p_{2} , \ldots ,p_{N} } \right]^{T}$. Finally, the RIS uses the codebook ${{\varvec{\uppsi}}}_{{n^{ * } }}$ where $n^{ * } = \arg {\kern 1pt} \,\mathop {\max }\limits_{n} \left[ {{\mathbf{p}}\left( {s^{\prime}} \right)} \right]_{n}$.

The key advantage of the proposed scheme is that instead of using the rates as the deep neural network outputs, we use the pull-probability vector ${\mathbf{p}}\left( s \right) = \left[ {p_{1} \left( T \right),p_{2} \left( T \right), \ldots ,p_{N} \left( T \right)} \right]^{T}$ computed at step 4 using the updated weights which are in turn computed using the normalized reward (step 7). The normalized reward $r_{{n_{t} }}$ is computed using the received rate $R_{{n_{t} }} \left( s \right)$ as $r_{{n_{t} }} = {{R_{{n_{t} }} \left( s \right)} \mathord{\left/ {\vphantom {{R_{{n_{t} }} \left( s \right)} {R_{\max } }}} \right. \kern-\nulldelimiterspace} {R_{\max } }}$, where $R_{\max }$ is the maximum achievable rate or a large number to make sure that $r_{{n_{t} }} \in \left[ {0,1} \right]$.

#### *Note on the complexity in comparison with the base method in *[26]

**Fig. 3:**
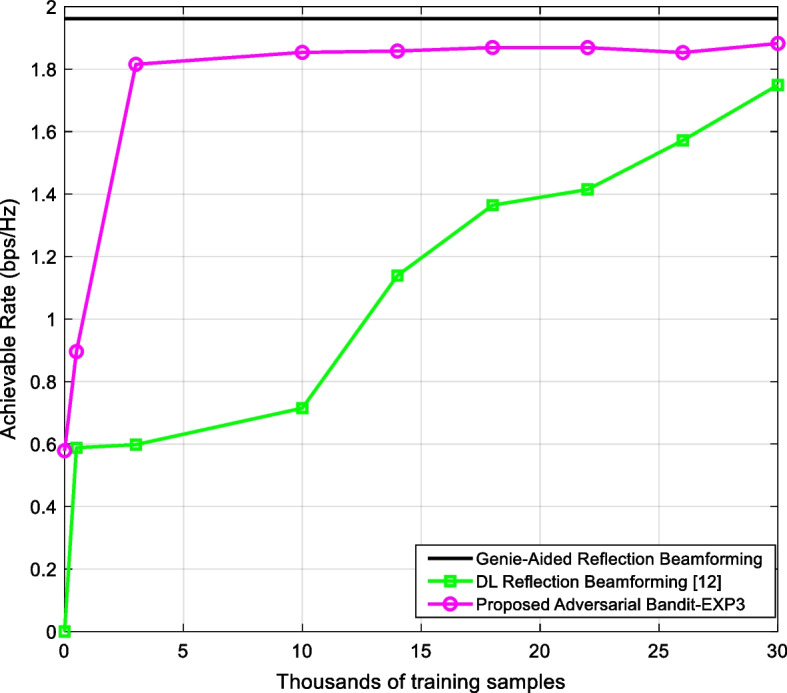
The achievable rate versus the number of training data of the proposed EXP3-based scheme in comparison with the reference DL reflection beamforming [26] and the reference genie-aided method (that assumes perfect knowledge of the channel) where $\overline{M} = 4$

The computation complexity of the learning phase of the proposed scheme in Table 1, is divided into two parts. The first part comprises steps 3–10 while the second part consists of step 12 which entails training a DL model. The main differences between the proposed method and the reference method [26] are as follows: During part 1, the proposed method involves extra *T* (where *T* is the number of the EXP algorithm iteration which is set to the size of the codebook $N = \left| P \right|$) basic scalar operations (such as multiplications, division, and exponentiation) whereas the reference method does not involve any operation at this stage. However, in part 2 where a DL model is trained, the reference method requires an order of magnitude (more than 10 times) larger dataset to reach similar achievable rate (c.f. Fig. 3). The training of a DL model is by far the most dominant computation burden compared to the first part.

#### Note on the EXP algorithm’s hyperparameters values

So far $\gamma \in \left( {0,1} \right]$ and the number of iteration *T* are set manually to 0.1 and $N = \left| P \right|$ where we have noticed a substantial gain in the required training dataset size. However, further investigation is required to infer the optimal values of these hyperparameters over varying system parameters. One approach is to perform an exhaustive grid search over all possible values. The optimal values would be the ones that provide the best achievable rate and/or spectral energy efficiency at the constrain of an order of magnitude smaller training dataset. Another very appealing approach is to resort to meta-learning to learn these hyperparameters using for instance model-agnostic meta-learning (MAML) algorithm [44, 45].

## Results and discussion

The proposed EXP3-based learning scheme is evaluated using the outdoor ray tracing scenario O1 from the deep-MIMO dataset that is publicly available at [39]. For the sake of facilitating the comparison, a similar setup is used in [26] as well (see Fig. 2). The results herein are also validated using channel data generated using SimRIS tool [2]. The adopted RIS employs a uniform planar array (UPA) with 16-by-16 (*M* = 256) antenna elements with 3 dBi gain at the 28 GHz mmWave setup. The transmit power is set to 10 dBW while the receiver’s noise figure is 5 dB. The codebook $P$ is constructed using a 2D discrete Fourier transform (DFT) matrix.

The number of subcarriers involved in ${\overline{\mathbf{h}}}\left( s \right)$ is $\left( {\overline{K} = 64} \right) \ll \left( {K = 512} \right)$, which sets the input of the DL model equal to $2\overline{K}\overline{M}$. The sampled channel vector is normalized prior to the training phase. The DL models consists of four layers similar to the one used in [26] where the number of the nodes in the hidden layers is $\left( {2\overline{K}\overline{M},4M,4M,M} \right)$. The regular training and optimization parameters are: batch size set to 500 samples, dropout rate is 0.5, and L_2_ regularization factor is 0.0001. Of course, we do not attempt to optimize the DL model but we will discuss its quality using state-of-the-art techniques such as the power low exponents [40] in section IV.

**Fig. 4:**
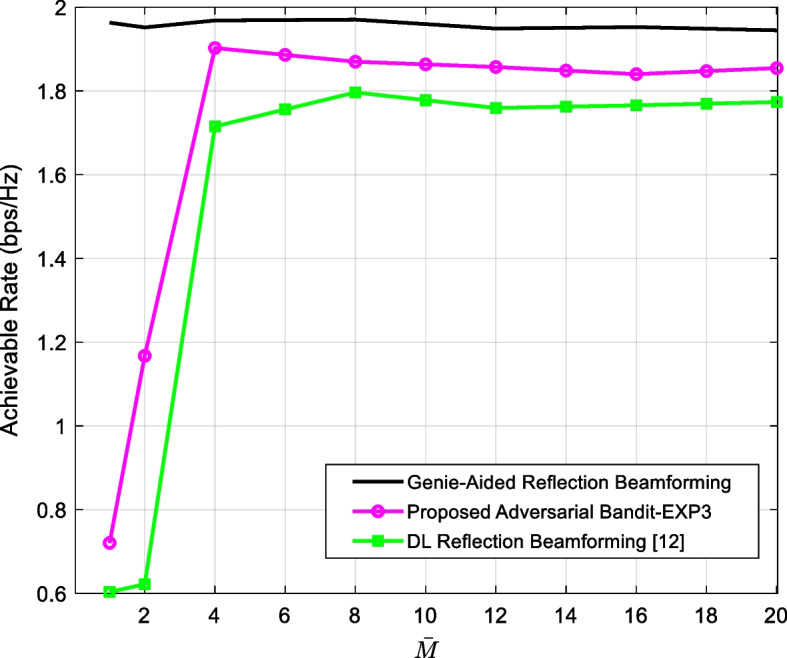
The achievable rate versus the number of active elements of the proposed EXP3-based scheme in comparison with the reference DL reflection beamforming [26] and the reference genie-aided method (that assumes perfect knowledge of the channel) where the number of training data is set to 30 K

Figure 3 shows the achievable rate as a function of the number of training samples. The proposed EXP3-based scheme requires substantially less data as compared to the DL reflection beamforming technique [26], owing to the optimal selection of the dataset which stresses that less likely reflection beams are given lower probability, excluding them during the exploitation phase of the EXP3 algorithm (Table 1, Step 5). The reference DL reflection beamforming requires more active elements $\overline{M}$ to sustain competitive performance as shown in Fig. 4 where EXP3-based learning schemes achieves 96% of the optimal achievable rate compared to 88% using the reference method in [26]. However, this will come at the expense of higher power consumption. Nevertheless, it seems that as far as the number of active elements is higher than 4, all methods are showing close to the performance of the genie-aided method.

**Fig. 5:**
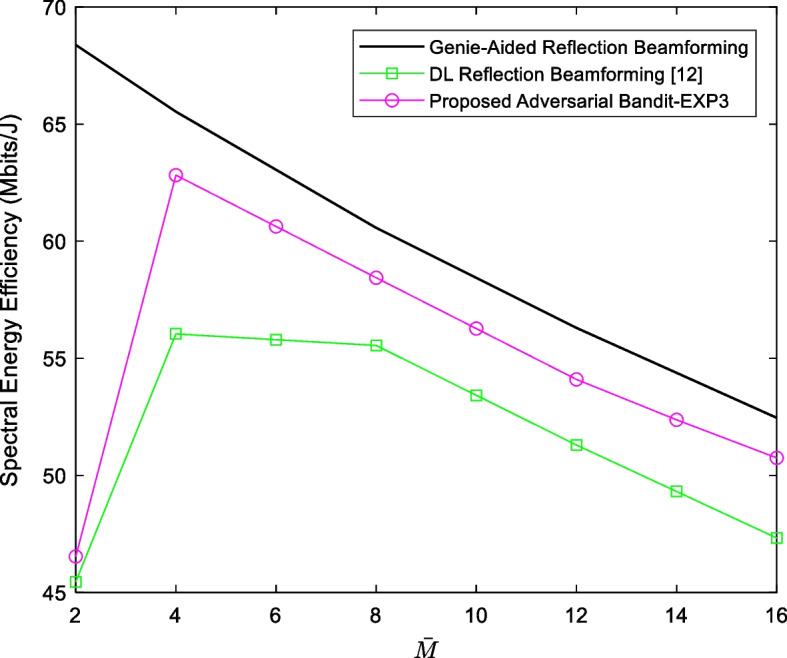
The energy efficiency $\eta$ versus the number of active elements $\overline{M}$ of the proposed EXP3-based scheme in comparison with the reference DL reflection beamforming [26] and the reference genie-aided method where the number of training data is set to 30 K

We reformulate the energy efficiency as $\eta = {{W \times R} \mathord{\left/ {\vphantom {{W \times R} {P_{c} }}} \right. \kern-\nulldelimiterspace} {P_{c} }}$ measured in Mbit/J, where $W$ is the transmission bandwidth and $P_{c}$ is the RIS power consumption which can be broken down to

4 $$P_{c} = {\text{MP}}_{{{\text{PS}}}} + \overline{M}\left( {P_{{{\text{LNA}}}} + P_{{{\text{RF}}}} + 2{\text{FOM}}_{{\text{W}}} f_{{{\text{FS}}}} 2^{b} } \right)$$

where the term $2{\text{FOM}}_{{\text{W}}} f_{{{\text{FS}}}} 2^{b}$ is the power consumption of a *b*-bits ADC with $f_{{{\text{FS}}}}$ being the Nyquist sampling frequency and ${\text{FOM}}_{{\text{W}}}$ is the Walden’s figure of merit [46]. $P_{{{\text{PS}}}}$, $P_{{{\text{LNA}}}}$ and $P_{{{\text{RF}}}}$ are, respectively, the power consumptions of the phase-shifter in the passive RF path, and the low-noise amplifier (LNA) and the rest of the RF chain along the active paths. As per the state-of-the-art RF parts’ specifications, these variables are set to $P_{{{\text{PS}}}} = 10\;{\text{mW}}$, $P_{{{\text{LNA}}}} = 20\;{\text{mW}}$, $P_{{{\text{RF}}}} = 40\;{\text{mW}}$ and the baseband processing power of 200 mW is assumed. Assuming similar values like the ones in [26] and [47], ${\text{FOM}}_{{\text{W}}} = 46.1\,f{\text{J/conversion}}$ at $W = 100\,{\text{MHz}}$ and $b = 4\,{\text{bits}}$. As such, Fig. 5 depicts the energy efficiency $\eta$ as a function of the number of active elements $\overline{M}$. Like the reference DL reflection BF [26], the proposed method shows optimal but higher energy efficiency performance using four active elements only.

In light of these results, the EXP3-based adversarial bandit method demonstrates outstanding performance gains compared to other state-of-the-art methods. So far, the adopted deep neural network architecture is similar to the one used in [26]. The reason being that one would be keen to see the effect of using a new learning scheme rather than proposing a new DL model. The other reason, which we discuss in the next section, is that one will also be interested to compare the quality of the two networks trained using $\Upsilon \leftarrow \left( {{\overline{\mathbf{h}}}\left( s \right),{\mathbf{r}}\left( s \right)} \right)$ for [26] and $\Upsilon \leftarrow \left( {{\overline{\mathbf{h}}}\left( s \right),{\mathbf{p}}\left( s \right)} \right)$ in the proposed method. However, let us first introduce another computationally efficient adversarial bandit-based scheme that uses the Follow the Perturbed Leader (FPL) algorithm.

## Improving and evaluation of the quality of proposed approaches 

Even if the EXP3 algorithm has efficient theoretical guarantees, it is computationally expensive due to the calculation of the exponential terms [42]. The FPL algorithm is then introduced to alleviate the burden by following the reflection beam that has the best performance while adding exponential noise to it to provide exploration [48]. Even though the baseline FPL algorithm does not have appreciated theoretical guarantees, it is worth evaluating its performance in the scope of the current RIS refection beamforming prediction. Table 2 shows the FPL algorithm where the exponential noise, which can be computed offline, is added in step 4 to provide exploration.

**Table 2:**
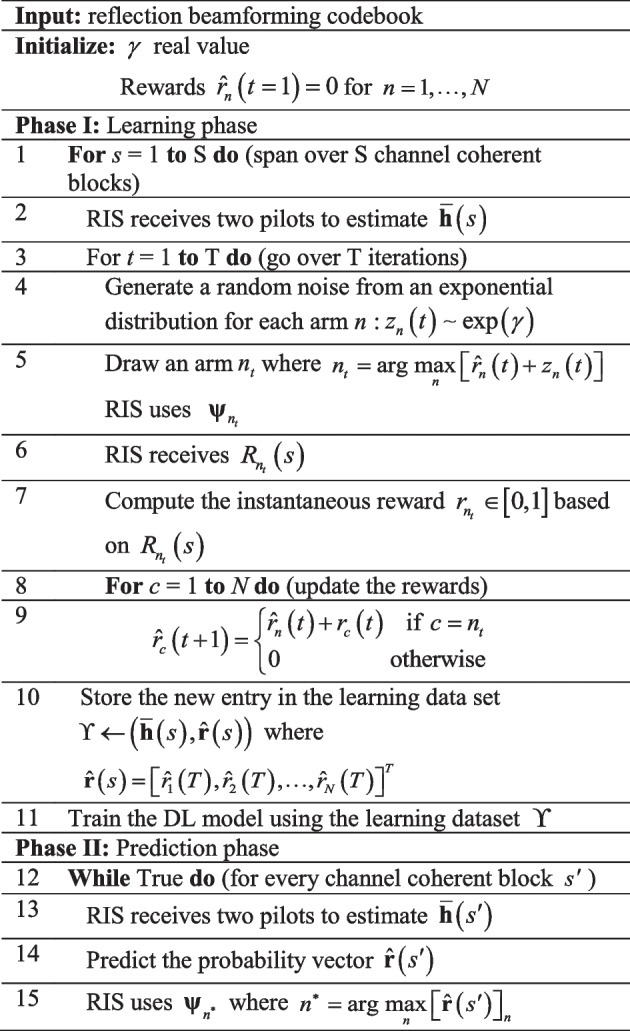
Follow the perturbed leader (FPL) scheme for reflection beamforming perdition

**Fig. 6:**
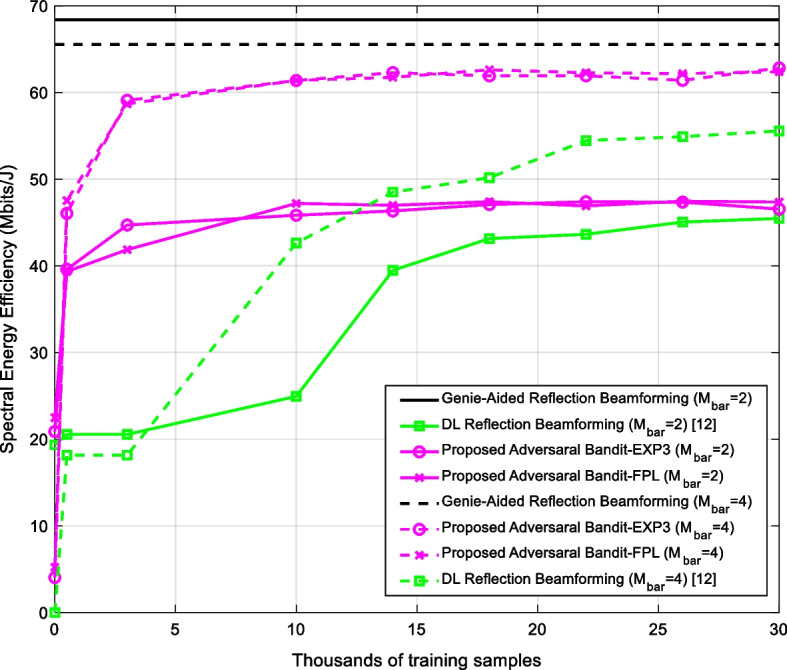
The spectral energy efficiency versus the number of training samples and the number of active elements $\overline{M}$ for the proposed FPL-based scheme

**Fig. 7:**
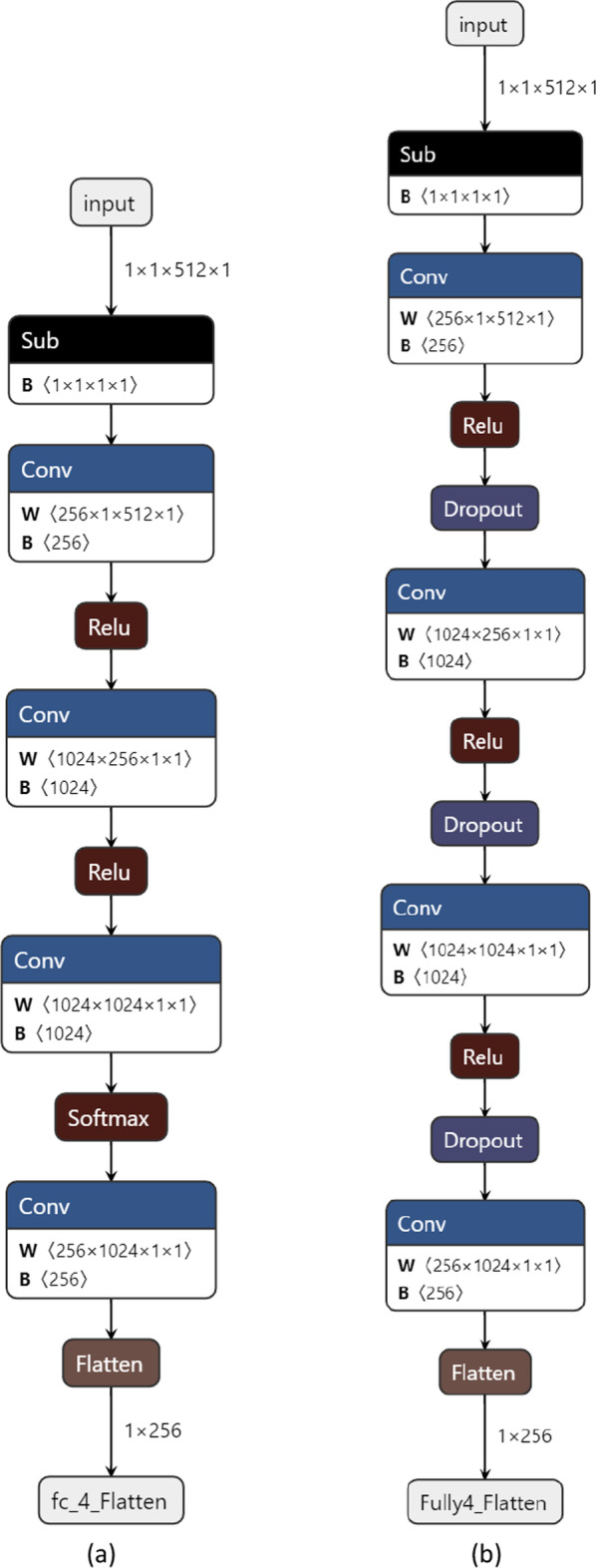
The DL models used with **a** EXP3/FTPL algorithm and with **b** the reference method [26] generated using Netron

Figure 6 shows that the FPL algorithm provides similar performances to the EXP3 algorithm at the expense of less “explainability” information, such as the pull-probabilities and weights inherent in EXP3. However, how one can decide which algorithm is better beyond just comparing the achievable rates (accuracy)? Even if all algorithms have different approaches to build the training dataset $\Upsilon$, they all share a similar model. Figure 7,^5^ depicts the DL models used with the EXP3/FPL algorithms and the reference method [26]. The slit differences are in using the dropout layers to improve the regularization of the reference method and the use of the softmax activation for the model used with EXP3 to generate the pull-probabilities. Nevertheless, in the end, these models are considered as black boxes that need to be compared.

**Fig. 8:**
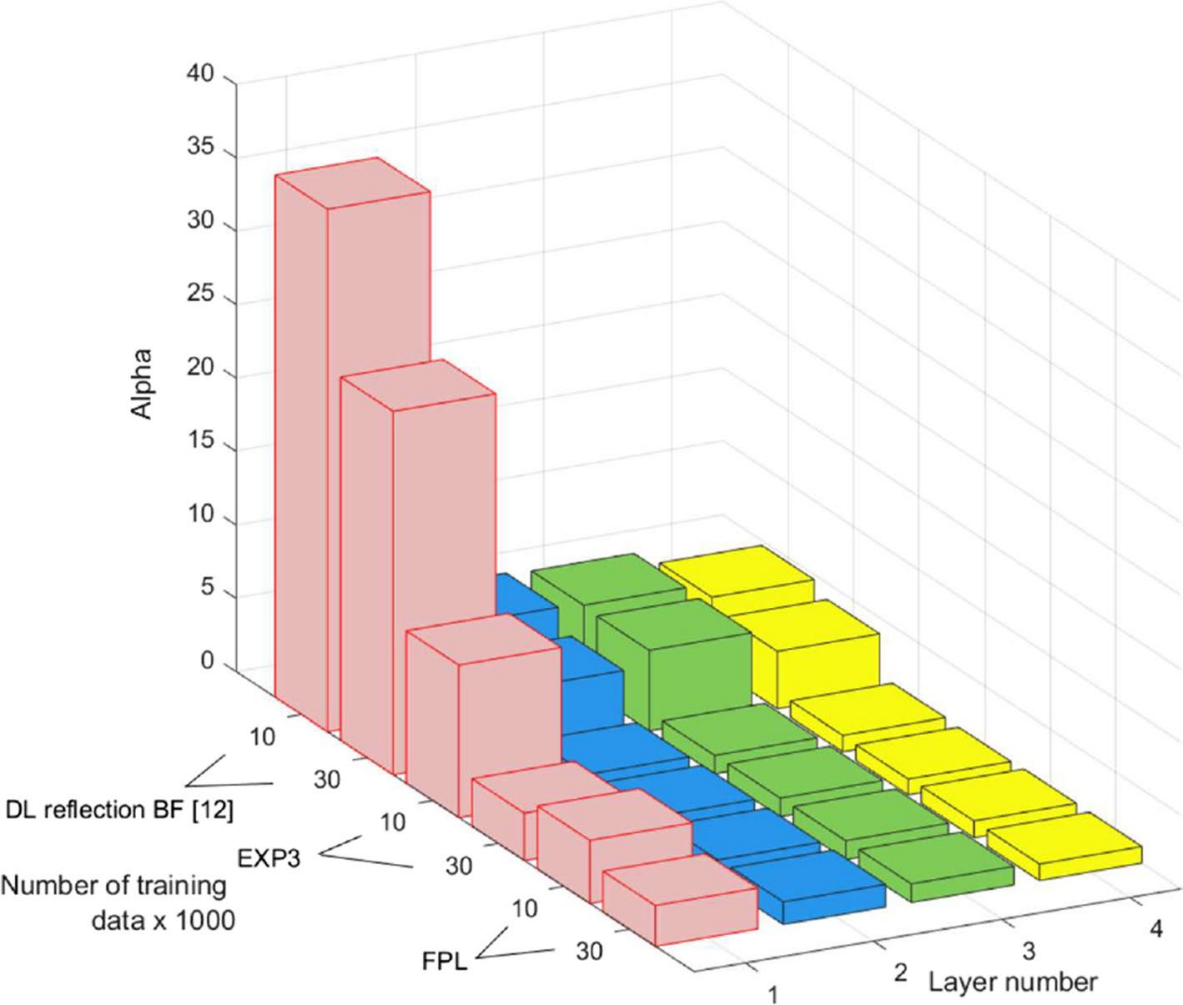
The power low (PL) exponent *α* to compare the quality of the models used in the reference method [26], EXP3 and FLP algorithms.

It is beyond the scope of this paper to dig into explainability of DL models which can be found in [49]. We will rather use state-of-the-art tools from [40] and mainly power low (PL) exponents to compare the quality of the DL models. Figure 8 depicts the PL exponents for the four layers. Indeed, we expect that a poorly trained model will lack good (i.e., small exponents *α*) PL behavior in some layers, whereas the EXP3 has, on average, smaller *α* values than the reference method, with all *α* ≤ 6 and with smaller mean/median *α*. It also has far fewer unusually large outlying *α* values than the reference method. The model used with FPL algorithm is rather showing the best training quality at the expense of less theoretical guarantees. The exponent values are obtained using the WeightWatcher tool from [50]. For future investigation, this should also be contrasted with the behavior displayed by scale-dependent metrics such as the Frobenius norm and the Spectral norm [40].

## Conclusion

RIS-aided communication has received increasing interest from the research community, with discussions not just about its unprecedented potential but also about the stumbling blocks with regard to feasible real-time operation. Among others, channel estimation overhead is regarded as a serious issue, which makes the adoption of DL tools an attractive alternative to solve the problem. As such, we have discussed two adversarial bandit-based schemes that provide substantial spectral and energy efficiency gains. We have also discussed the associated DL models’ quality using the PL exponents to show the training quality using the dataset generated from the proposed schemes. Our work contributes to shedding light on the potential improvements that can be made in exploring the interplay between ML and RISs. For future research, one could investigate the proposed schemes under different channel and system parameters while adopting meta-learning approach [33, 51], to improve the online training performance. Different DL models can also be used along with these schemes wherein explainability shall be given a considerable attention [49] to improve the trustworthiness of the DL-enabled solutions. Last but not least, at the hardware level, one can use low power root-mean-square and envelop detectors to capture the high dimensional received signal features along space (over RIS geometry) and time so that more advanced DL models such as long short-term memory (LSTM) model can be leveraged.

## Data Availability

Data sharing is not applicable to this article as no datasets were generated during the current study. All data generated or analyzed during this study are included in this article.

## Abbreviations

5G: Fifth-generation
6G: Sixth-generation
PHY: Physical layer
MIMO: Multiple-input multiple-output
RIS: Reconfigurable intelligent surfaces
SISO: Single-input single-output
RAN: Radio access network
MAC: Medium access control
DL: Deep learning
RNN: Recurrent neural network
OFDM: Orthogonal frequency division multiplexing
CNN: Convolutional neural network
DRL: Deep reinforcement learning
EXP3: Exponential-weight algorithm for exploration and exploitation
FPL: Follow the perturbed leader
PL: Power low
RF: Radio frequency
LNA: Low-noise amplifier
LSTM: Long short-term memory

## Footnotes

1 Herein, the transmitter mimics the base-transceiver station while the receiver mimics the user equipment.

2 When using DL tools, it is hard to evaluate the merits and the performance of the proposed methods in comparison with reference methods unless similar models and datasets are used. Otherwise, any performance gain may be attributed to system and channel model and dataset differences.

3 The benefit of RIS is mostly harnessed when the direct path is blocked or simply very weak. Such an assumption helps in simplifying the analysis of the algorithm [1 2,26,39].

4 So far only discrete phase variation is considered. To achieve better achievable rate both the phase and the amplitudes of the RIS’ elements shall be controlled at the expense of increasing the search space for the optimal solution.

5 Netron is used to generate Fig. 7. It is a visualizer for neural network, deep learning, and machine learning models. It can be acquired from https://www.electronjs.org/apps/netron.
